# Diversin Is Overexpressed in Breast Cancer and Accelerates Cell Proliferation and Invasion

**DOI:** 10.1371/journal.pone.0098591

**Published:** 2014-05-23

**Authors:** Xinmiao Yu, Minghao Wang, Qianze Dong, Feng Jin

**Affiliations:** 1 Department of Surgical Oncology and Breast Surgery, First Affiliated Hospital of China Medical University, Shenyang, China; 2 Department of Neurosurgery, First Affiliated Hospital of China Medical University, Shenyang, China; 3 Department of pathology, First Affiliated Hospital of China Medical University, Shenyang, China; II Università di Napoli, Italy

## Abstract

Diversin was recently reported to play roles in Wnt and JNK pathways. However, the expression pattern and biological roles of diversin in human breast cancer have not been reported. In the present study, we found that diversin was overexpressed in breast cancer specimens by immunohistochemistry and western blot. Significant association was observed between diversin overexpression and TNM stage (p = 0.0036), nodal metastasis (p = 0.0033), negative estrogen receptor expression (p = 0.0012) and triple-negative status (p = 0.0017). Furthermore, colony formation assay and matrigel invasion assay showed that knockdown of diversin expression in MDA-MB-231 cell line with high endogenous expression decreased cell proliferation and cell invasion. Transfection of diversin plasmid in MCF-7 cell line increased cell proliferation and invasion. Further analysis showed that diversin depletion downregulated JNK phosphorylation while its overexpression upregulated JNK phosphorylation. In conclusion, our study demonstrated that diversin was overexpressed in human breast cancers. Diversin could contribute to breast cancer cell proliferation and invasion.

## Introduction

Breast cancer survival has improved significantly over the last decades however, it still ranks second among cancer deaths in women [1]. Exploring new bio-markers to predict tumor progression and potential target therapies is important [2]–[5].

Diversin is an ankyrin repeat protein, has been implicated in both the canonical and non-canonical Wnt pathways [6], [7]. Diversin protein contains an N-terminal domain that contains eight ankyrin repeats, the central domain which interacts with CKIε and the C-terminal domain interacts with Conductin or Axin [7]. Diversin could regulate various morphogenetic events, including the convergent extension in gastrulation and heart formation during zebrafish development and vascular development in cell culture [8]. Diversin localizes to the centrosome in mammalian cells [9]. The centrosome and its derivative cilium play important roles in many of signaling pathways such as Hedgehog and Wnt [10], [11]. In addition, Diversin could translocate into the nucleus, where it interacts with the transcription factor AF9 and activates JNK pathway [12].

The fact that diversin participates in Wnt pathway and regulates cell morphology and several signaling pathways indicates its potential roles on biological behavior of cancer cell. To data, the expression pattern and biological roles of diversin in human breast cancer remains unclear. In the present study, we examined the expression of diversin in 159 cases of breast cancer tissues and its relationship with the clinicopathological parameters. We also examined the biological roles of diversin in breast cancer cell lines and explored its potential mechanism.

## Materials and Methods

The study protocol was approved by the institutional reviewer board of China Medical University. All participants provided their written informed consent and the investigation was conducted according to the principles expressed in the Declaration of Helsinki.

### Specimens and Immunohistochemistry

Tumor specimens were obtained from 159 patients diagnosed with invasive ductal carcinoma (IDC) who underwent resection in the First Affiliated Hospital of China Medical University between 2007 and 2011.

Surgically excised tumor specimens were fixed with 10% neutral formalin and embedded in paraffin, and 4- µm-thick sections were prepared. Immunostaining was performed using the avidin–biotin–peroxidase complex method (Ultrasensitive™, MaiXin, Fuzhou, Chin . The sections were deparaffinized in xylene, rehydrated with graded alcohol, and then boiled in 0.01 M citrate buffer (pH 6.0) for 2 min in an autoclave. Hydrogen peroxide (0.3%) was applied to block endogenous peroxide activity and the sections were incubated with normal goat serum to reduce nonspecific binding. Tissue sections were incubated with diversin mouse monoclonal antibody (1∶100 dilution; santa cruz). Mouse immunoglobulin (at the same concentration of the antigen specific antibody was used as a negative control. Staining was performed at 4°C overnight. Biotinylated goat anti-rabbit serum IgG was used as a secondary antibody. After washing, the sections were incubated with streptavidin–biotin conjugated with horseradish peroxidase, and the peroxidase reaction was developed with 3,3′-diaminobenzidine tetrahydrochloride. Counterstaining with hematoxylin was performed and the sections were dehydrated in ethanol before mounting.

Two independent blinded investigators examined all tumor slides randomly. Positive nuclear/cytoplasmic staining was considered positive. Immunostaining of diversin was scored on a semi-quantitative scale by evaluating staining intensity and percentage. We calculated the percentage of positively stained cells. The staining intensity was categorized as follows: 0, negative; 1, moderate; and 2, strong. The staining percentage of tumor specimens was scored as 0, 0%; 1 1–5%; 2, 6–25%; 3, 26–75% and 4, 76–100%. The scores of each tumor sample were multiplied to give a final score of 0 to 8, and the tumor samples with a final score of 4–8 were finally determined as diversin overexpression.

### Cell culture and transfection

MCF-7 and MDA-MB-231 cell lines were obtained from American Type Culture Collection (Manassas, VA, USA). The cells were cultured in DMEM (Invitrogen, Carlsbad, CA, USA) containing 10% fetal calf serum (Invitrogen), 100 IU/ml penicillin (Sigma, St. Louis, MO, USA), and 100 µg/ml streptomycin (Sigm . Cells were grown on sterilized culture dishes and were passaged every 2 days with 0.25% trypsin (Invitrogen).

DharmaFECT1 reagent was used for siRNA transfection (Qiagen, Chicago, IL, USA) according to the manufacturercs instructions. The protein level was assessed 48 h later by western blotting. Diversin-siRNA was synthesized by GenePharma Co, Ltd (Shanghai, Chin . The sequences of the four double-stranded oligonucleotides were as follows: 5' GAGGCACUCAAACUAAGAATT 3', 5' UUCUUAGUUUGAGUGCCUCTT 3'.

Attractene reagent was used for plasmid transfection (Qiagen, Hilden, Germany) according to the manufacturer’s instructions. pcDNA3.1-HA-Diversin plasmid was a gift from Dr Walter Birchmeier (Max Delbrueck-Center for Molecular Medicine, Berlin, Germany).

### Western blot analysis

Total proteins from cells were extracted in lysis buffer (Pierce, Rockford, IL) and quantified using the Bradford method. Samples of 50 µg of protein were separated by SDS-PAGE. Samples were transferred to polyvinylidene fluoride membranes (Millipore, Billerica, MA, USA) and incubated overnight at 4°C with antibody against diversin (1∶500, Santa cruz), p-JNK, JNK, p-p38, p38 (1∶1000; Cell signaling, Boston, MA, USA) and a mouse monoclonal antibody against GAPDH (1∶1000; Santa Cruz). After incubation with peroxidase-coupled anti-mouse/rabbit IgG (Santa Cruz) at 37°C for 2 h, bound proteins were visualized using ECL (Pierce) and detected using a BioImaging System (UVP Inc., Upland, CA, USA).

### Quantitative real-time PCR (SYBR Green method)

Total RNA was extracted from cells using Trizol(Qiagen). Reverse transcription of 1 µg of RNA was done using the high capacity cDNA RT kit (Applied Biosystems) following the manufacturer's instructions.

Quantitative real-time PCR was done using SYBR Green PCR master mix (Applied Biosystems) in a total volume of 20 µl on 7900HT fast Real-time PCR system (Applied Biosystems) as follows: 50°C for 2 min, 95°C for 10 min, 40 cycles of 95°C for 15 sec, 60°C for 60 sec. The sequences of the primer pairs are as follows: Diversin forward, 5' TGCGTACAAAGGCCAAACAGAG 3', Diversin reverse, 5' CAGCAAGATCTGGACCACAGGA 3'; β-actin forward, 5' ATAGCACAGCCTGGATAGCAACGTAC 3', β-actin reverse, 5' CACCTTCTACAATGAGCTGCGTGTG 3'. A dissociation procedure was performed to generate a melting curve for confirmation of amplification specificity. β-actin was used as the reference gene. The relative levels of gene expression were represented as ΔCt = Ct gene –Ct reference, and the fold change of gene expression was calculated by the 2-ΔΔCt Method. Experiments were repeated in triplicate.

### Colony formation assay

For colony formation assay, cells were transfected for 48 h and then plated into three 6-cm cell culture dishes (1000 per dish) and incubated for 12 days. Plates were washed with PBS and stained with Giemsa. The number of colonies with more than 50 cells was counted. The colonies were manually counted using a microscope.

### Matrigel invasion assay

Cell invasion assay was performed using a 24-well Transwell chamber with a pore size of 8 µm (Costar, Cambridge, M . The inserts were coated with 20 µl Matrigel (1∶3 dilution, BD Bioscience, San Jose, CA, USA). 48 hours after the transfection, cells were trypsinized and 3×10^5^ cells in 100 µl of serum-free medium were transferred to the upper Matrigel chamber and incubated for 16 hours. Medium supplemented with 15% FBS was added to the lower chamber. After incubation, the non-invaded cells on the upper membrane surface were removed with a cotton tip, and the cells that passed through the filter were fixed with 4% paraformaldehyde and stained with hematoxylin. The numbers of invaded cells were counted in 3 randomly selected high power fields under microscope.

### Statistical analysis

SPSS version 11.5 for Windows was used for all statistical analyses. Chi-Square test was used to evaluate possible correlations between diversin overexpression and clinicopathologic factors. Student’s t-test was used to compare data between control and transfected cells. All p values were based on the two-sided statistical analysis and p<0.05 was considered to be statistically significant in difference.

## Results

### 1 Expression and distribution of diversin in breast cancer specimens

**Figure 1:**
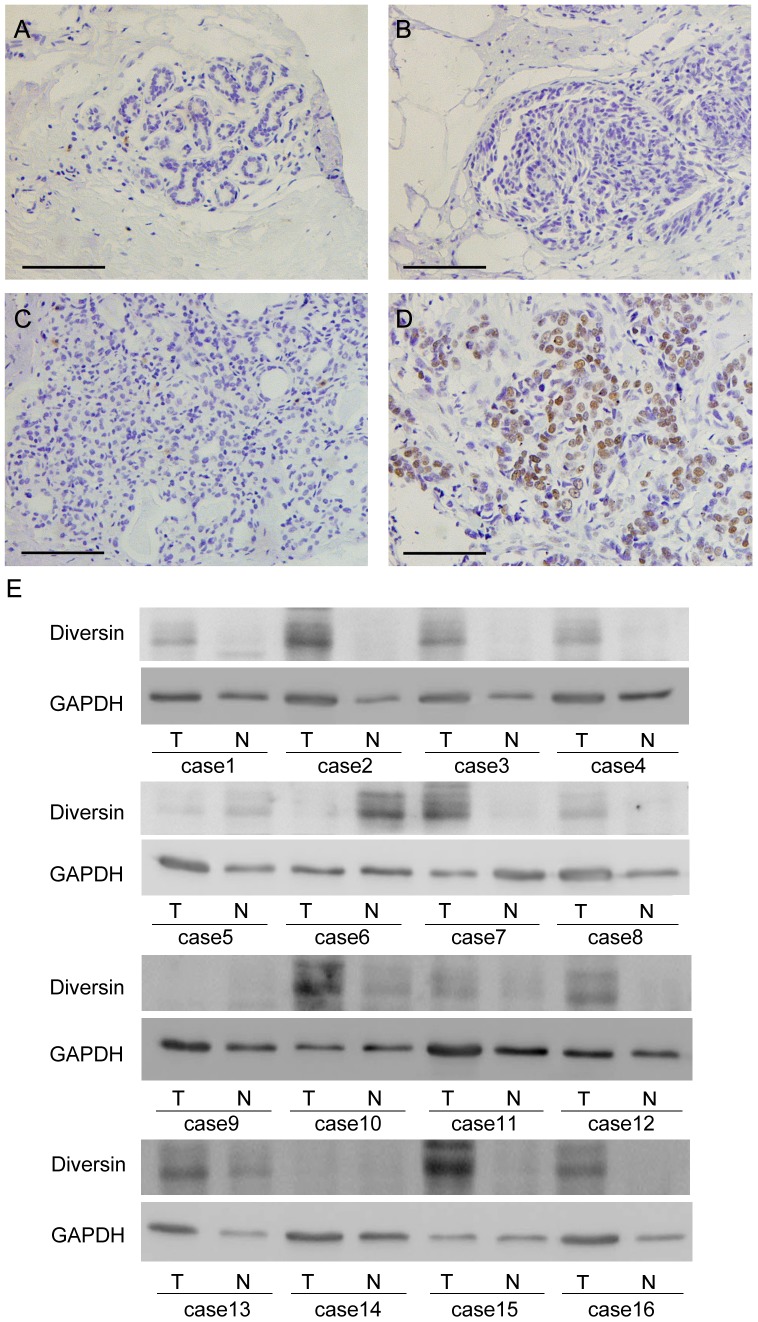
Expression pattern of diversin protein in breast cancer tissues. A. Negative diversin staining in the majority of normal breast tissue. B. Negative diversin staining in a case of ductal carcinoma in situ. C. Negative cytoplasmic staining of diversin in invasive ductal carcinoma. D. Strong nuclear staining of diversin in invasive ductal carcinoma. (Magnification, 200X) (Scale bar, 200 µm). E. Western blot analysis of diversin expression in 16 pairs of breast cancer and corresponding normal tissues.

A panel of 159 primary breast cancer samples as well as normal mammary gland was studied by immunohistochemistry and we found that in breast cancer specimens, diversin protein was mainly localized in the nuclear compartment. Negative diversin expression was detected in normal breast tissue (Figure1 . Negative/Weak cytoplasmic expression was found in 3 cases of ductal carcinoma in situ (DCIS) (Figure1B). Negative control using immunoglobulin showed negative staining. We found overexpression of diversin in 58 out of 159 (36.5%) invasive ductal carcinoma (IDC) specimens. Weak/Negative diversin staining was considered as normal expression (Figure1C). Strong nuclear staining with a final score≥4 was considered as diversin overexpression (Figure1D).

The relationship between the diversin expression and the clinical parameters was analyzed. As shown in Table 1, no statistical difference was found between the diversin overexpression and age (p = 0.9263), tumor size (p = 0.2371), ErbB2 status (p = 0.4613) and progesterone receptor status (p = 0.1054). Diversin overexpression correlated with advanced TNM stage (p = 0.0036), positive nodal metastasis (p = 0.0033) and negative estrogen receptor status (p = 0.0012). The diversin overexpression rate in stage III and II breast cancers (45.3%) was higher than stageI (22.5%) breast cancers. In addition, in the 38 cases of triple-negative breast cancer cases, there were 22 cases with positive diversin staining (57.9%), which was significantly higher than non-triple-negative cases (29.7%) (p = 0.017).

**Table 1:** Distribution of diversin status in breast cancer according to clinicopathological characteristics.

Characteristics	Number of patients	diversin negative	diversin positive	*P*
Age				
<60	124	79	45	0.9263
≥60	35	22	13	
TNM stage				
I	62	48	14	0.0036
IIIII	97	53	44	
Tumor size				
<2 cm	56	39	17	0.2371
≥2 cm	103	62	41	
Lymph node metastasis				
Absent	82	61	21	0.0033
Present	77	40	37	
Estrogen receptor				
Absent	59	28	31	0.0012
Present	100	73	27	
Progesterone receptor				
Absent	77	44	33	0.1054
Present	82	57	25	
ErbB-2				
Absent	118	73	45	0.4613
Present	41	28	13	
Triple-negative				
Absent	121	85	36	0.0017
Present	38	16	22	

We also used western blot to detect diversin protein expression in freshly isolated breast cancer specimens. As shown in Figure E, Diversin protein expression levels were significantly higher in tumor tissues compared to corresponding normal tissues.

### 2. Diversin regulates breast cancer cell proliferation, invasion and JNK phosphorylation

**Figure 2:**
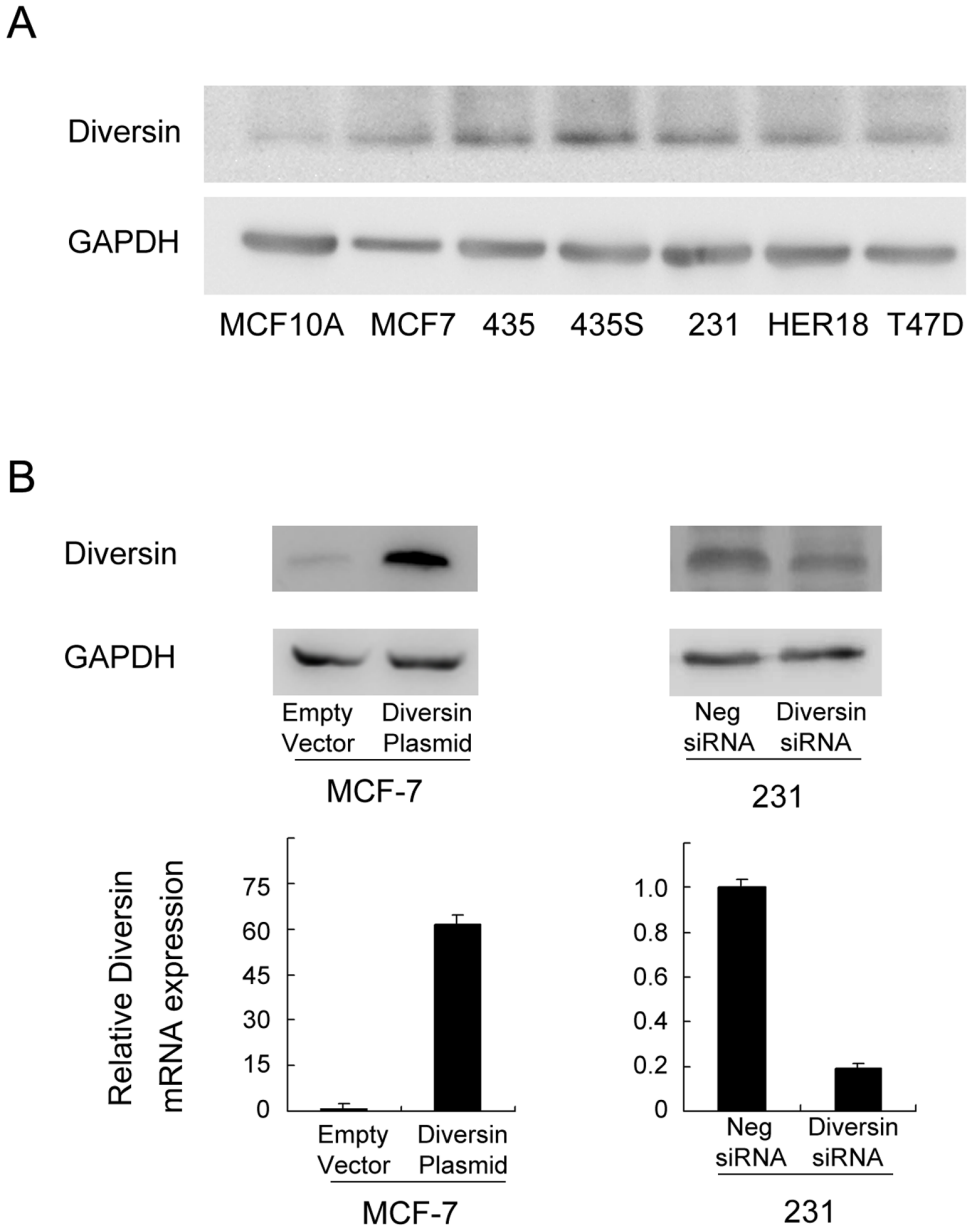
Diversin expression in breast cancer cell lines. A. Western blot analysis of diversin expression in breast cancer cell lines Her-18, MDA-MB-231, MDA-MB-435, T46D, MCF-7 and normal breast cell line MCF-10A. B. Western blot and realtime PCR analysis showed that diversin siRNA treatment markedly decreases diversin mRNA and protein levels in MDA-MB-231 (p<0.05). Transfection of diversin plasmid significantly upregulated its expression in MCF-7 cell line (p<0.05).

**Figure 3:**
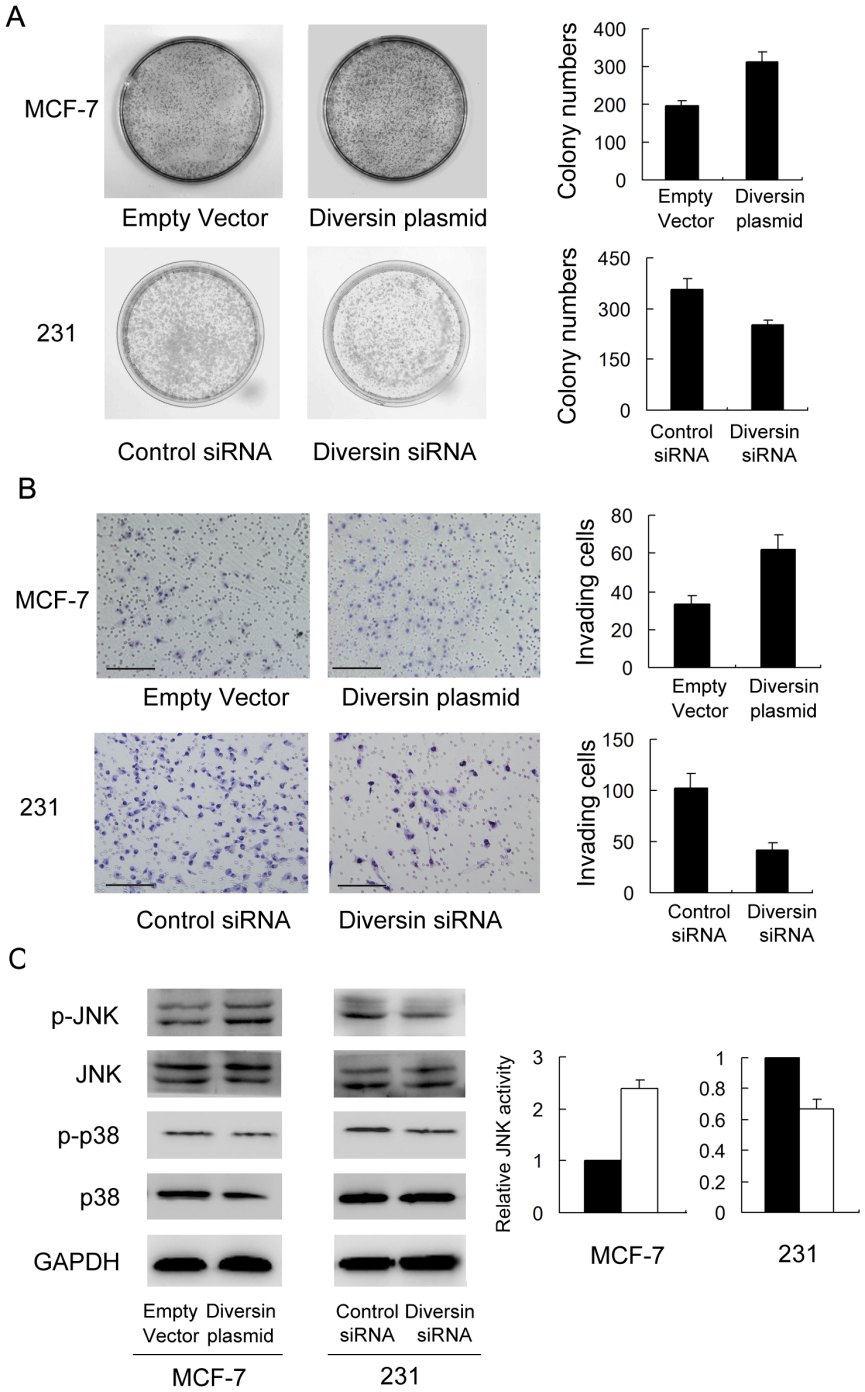
Diversin depletion inhibited and its overexpression promoted cell proliferation and invasion and JNK phosphorylation. A. Colony formation assay showed that diversin knockdown inhibited proliferation in MDA-MB-231 cell line and its overexpression promoted proliferation in MCF-7 cell line. B. Diversin depletion decreased the level of 231 cell invasion and its overexpression facilitated invasion in MCF-7 cells. C. Western blotting revealed that knockdown of diversin decreased the levels of p-JNK and diversin transfection increased p-JNK, without significant changes of total JNK, p-p38 and p38. Scale bar, 200 µm.

We examined diversin expression levels in normal breast ductal cell line MCF-10A and six breast cancer cell lines by western blot. We found low diversin expression in MCF-10A, MCF-7 cell lines and high levels of diversin in MDA-MB-231 cell lines, which were derived from triple-negative breast cancers (Figure 2A). We employed siRNA in MDA-MB-231 cell line to deplete diversin expression. Diversin plasmid transfection was performed in MCF-7 cell line. Western blot and realtime PCR analysis confirmed the diversin knockdown and transfection effeciency (Figure 2B). The proliferation rate was determined by colony formation assay. We found that treatment of diversin-specific siRNA resulted in a decrease in colony formation ability compared with control siRNA (231 Con vs siRNA: 357±31 vs 251±17) (p<0.05). Diversin overexpression increased colony formation ability (MCF-7 Con vs siRNA: 197±12 vs 314±25) (p<0.05) (Figure 3A). Matrigel invasion assay was employed to characterize the role of diversin on cell invasion. As shown in Figure 3B, a significant decrease of cell invasion (231: 58.8%) was observed in cells with diversin knockdown compared with scramble controls and diversin overexpression promoted MCF-7 cancer cell invasion (87.8%). It was reported that diversin was involved in JNK activation, which has been shown to regulate cancer cell invasion. To explore the potential mechanism of diversin on the biological behavior of cancer cells, we explored the effect of diversin knockdown and overexpression on JNK and p38 pathway activation. As shown in Figure? 3C, knockdown of diversin decreased the protein levels of p-JNK in MDA-MB-231 cells while its overexpression increased JNK phosphorylation in MCF-7 cells. The change of p38 phosphorylation was not significant. These results suggested that diversin regulated cell invasion by JNK pathway modulation.

## Discussion

The reports showing diversin participates in canonical and non-canonical Wnt pathway and its regulation of cell migration and proliferation indicate its potential roles on cancer cell invasion and metastasis [6], [8], [12]. However, its expression pattern and biological roles in breast cancer have not been examined. We demonstrated that the level of diversin in invasive ductal carcinoma was higher than that in normal breast tissues. Diversin overexpression was located in the nuclear compartment of cancer cells, which was in accord with previous reports [12]. There were correlations between diversin up-regulation and tumor stage, and nodal metastasis, suggesting diversin might associate with breast cancer progression. In addition, diversin overexpression correlated with negative estrogen receptor expression and triple-negative breast cancer. These results not only showed that diversin protein was overexpressed in breast cancers, but also suggested tumors with diversin overexpression were more malignant and less likely to respond to endocrine therapy [13]–[16].

To explore the biological roles of diversin on breast cancer cells, we blocked diversin function by using siRNA treatment in MDA-MB-231 triple-negative breast cancer cell line, which has high endogenous diversin expression. We found that diversin depletion caused an obvious decrease in the proliferation rate and invading ability. Diversin overexpression in MCF-7 cancer cell line increased its proliferation and invasion, which was in accordance with our immunohistochemical data. It was reported that nuclear located diversin expression could interact AF9 and drive JNK dependent gene expression [12]. In the present study, we found that knockdown of diversin decreased the protein levels of p-JNK and upregulation of diversin increased JNK activity, whereas the level of p-p38 exhibited no significant changes. JNK has been reported to potentiate oncogenesis depending upon the cellular context [17], [18]. JNK activation increased cell proliferation, migration, invasion and its depletion inhibited malignant behavior of cancer cells [19]–[21]. In addition, JNK activation was reported to induce MMP9 transcription [22], which is a well known invasion related protein. This result indicated that diversin regulated malignant biological behavior of breast cancer cell via JNK pathway modulation.

In conclusion, our study found that diversin overexpression existed in breast cancer and correlated with TNM stage and nodal status. Diversin contributed to the malignant cell growth and invading ability through regulation of JNK pathway. These results suggested that diversin functions as an important regulator of breast cancer progression.
