# Correction: Diversin Is Overexpressed in Breast Cancer and Accelerates Cell Proliferation and Invasion

**DOI:** 10.1371/journal.pone.0116045

**Published:** 2014-12-18

**Authors:** The PLOS ONE Staff

The affiliation for the fourth author is incorrect. Feng Jin is not affiliated with #2 but with #1 Department of Surgical Oncology and Breast Surgery, First Affiliated Hospital of China Medical University, Shenyang, China.
